# Long-Term Real-World Outcomes of Mavacamten in Symptomatic Obstructive Hypertrophic Cardiomyopathy up to 108 Weeks

**DOI:** 10.3390/jcm14228181

**Published:** 2025-11-18

**Authors:** Nosheen Reza, Anandkumar Dubey, Nadim Mahmud, Melissa A. Austin, Estherland Duqueney, Parth Patel, Ellen Boakye, Amy Marzolf, Nicole Hornsby, Alejandro de Feria, Thomas Carattini, Patricia Schuler, Anjali Tiku Owens

**Affiliations:** 1 Division of Cardiovascular Medicine, Department of Medicine, Perelman School of Medicine at the University of Pennsylvania, Philadelphia, PA 19104, USA; 2 Bristol Myers Squibb, Princeton, NJ 08540, USA; 3 Division of Gastroenterology, Department of Medicine, Perelman School of Medicine at the University of Pennsylvania, Philadelphia, PA 19104, USA; 4 NYU Langone Health, New York, NY 10016, USA; 5 Department of Medicine, Perelman School of Medicine at the University of Pennsylvania, Philadelphia, PA 19104, USA; 6 Division of Cardiovascular Medicine, University of Southern California Keck School of Medicine, Los Angeles, CA 90033, USA

**Keywords:** hypertrophic cardiomyopathy, mavacamten, cardiac myosin, echocardiography

## Abstract

**Background/Objectives**: Mavacamten is a first-in-class cardiac myosin inhibitor approved for the treatment of symptomatic obstructive hypertrophic cardiomyopathy (HCM). Long-term data regarding its real-world safety and effectiveness are limited. We aimed to describe the real-world experience of mavacamten in a large obstructive HCM cohort at a high-volume HCM center in the United States. **Methods**: Adult patients initiated on mavacamten between 29 April 2022 and 19 January 2025 at a single HCM center (*n* = 163) were retrospectively identified. Clinical effectiveness and safety data were collected through 108 weeks of treatment. **Results**: Rapid and sustained reductions in resting (baseline mean 53.0 ± 36.7 mm Hg to 10.0 ± 11.0 mm Hg) and Valsalva left ventricular outflow tract gradients (baseline mean 79.7 ± 33.2 mm Hg to 16.6 ± 15.4 mm Hg) were observed during treatment throughout the study period along with substantial improvements in New York Heart Association (NYHA) class (75% with >1 NYHA class improvement by Week 12). Mean maximal left ventricular wall thickness significantly decreased (β = 0.01 mm per week). Ten patients (6.1%) required temporary drug interruption due to decrement in left ventricular ejection fraction, and mavacamten was discontinued in six patients (3.7%). Doses of background beta blocker and nondihydropyridine calcium channel blocker were significantly reduced during the study period (*p* < 0.001). **Conclusions**: In this large single-center real-world experience of mavacamten therapy, mavacamten was highly effective and maintained an acceptable safety profile, comparable to the clinical trial long-term extension experience.

## 1. Introduction

Hypertrophic cardiomyopathy (HCM) is the most common inherited heart muscle disease with an estimated worldwide prevalence of 1:250–1:500 individuals [1]. Up to 75% of individuals with HCM have left ventricular outflow tract (LVOT) obstruction either at rest and/or with provocation, which can instigate and exacerbate left ventricular (LV) hypertrophy, adverse myocardial remodeling, myocardial ischemia [2], and pulmonary hypertension [3]. Moreover, LVOT obstruction is associated with an increased risk of heart failure (HF) and mortality [4]. Beyond this pathophysiology, LVOT obstruction is experienced by patients as chest pain, presyncope and syncope, exertional dyspnea, and effort intolerance; these symptoms significantly impair patients’ physical and social function and quality of life [5]. Historically, medical therapy for obstructive HCM was limited to therapies that were largely palliative and did not address the molecular mechanisms of disease.

The introduction of cardiac myosin inhibitors has transformed the landscape of HCM treatment. The first-in-class, selective, allosteric and reversible small-molecule cardiac myosin inhibitor mavacamten was shown to reduce myocardial hypercontractility and suppress hypertrophic and profibrotic gene programs in preclinical models [6]. In the subsequent two randomized, double-blinded, placebo-controlled phase 3 clinical trials, EXPLORER-HCM (NCT03470545) [5,7] and VALOR-HCM (NCT04349072) [8,9], patients treated with mavacamten experienced significant reduction in resting and provocable LVOT gradients; improvements in symptoms, functional class, exercise performance, cardiac biomarkers, and health status; and decreased eligibility for and progression to septal reduction therapy compared to placebo. In secondary and exploratory analyses, treatment with mavacamten has been shown to significantly improve measures of diastolic function including left atrial volume [10,11,12] and to reduce absolute intracellular myocardial mass index, LV mass index, and maximum LV wall thickness [11], demonstrating its potential as a disease-modifying therapy. These improvements in cardiac function and symptoms were sustained through 180 weeks in the open-label extension study MAVA-LTE [13].

Mavacamten is the first and only cardiac myosin inhibitor currently approved by several regulatory agencies worldwide as a treatment to improve functional capacity and symptoms in individuals with symptomatic New York Heart Association (NYHA) class II or III obstructive HCM. To date, published real-world experience studies have been limited in size and follow-up duration [14,15,16,17,18,19], leaving a considerable knowledge and practice gap regarding the longer-term clinical trajectory and management of real-world patients. As such, we aimed to report the real-world safety and effectiveness of mavacamten in a large cohort of patients with symptomatic obstructive HCM initiated on mavacamten at a high-volume HCM center in the 33 months post-U.S. Food and Drug Administration approval. Specific objectives were to describe functional and structural response as illustrated by changes in LVOT gradients, NYHA class, LV wall thickness, and left ventricular ejection fraction (LVEF) during treatment with mavacamten compared to baseline.

## 2. Materials and Methods

### 2.1. Study Design

We conducted a retrospective study of consecutive patients treated with mavacamten for symptomatic obstructive HCM at the Penn Center for Inherited Cardiovascular Disease, a comprehensive HCM center as described by the 2024 multisociety Guideline for the Diagnosis and Treatment of Patients With Hypertrophic Cardiomyopathy [1]. Patients eligible for inclusion were those aged > 18 years with NYHA class > II symptoms attributed to LVOT obstruction, LVEF > 55%, and peak LVOT gradient at rest or with provocation > 30 mmHg per U.S. Prescribing Information [20]. Patients included in the cohort were all those initiated on mavacamten from 29 April 2022 to 19 January 2025 with the exception of those who were previously enrolled in phase 3 mavacamten clinical trials. The study protocol was approved by the University of Pennsylvania Institutional Review Board (protocol number 857587). This study was performed in accordance with the Helsinki Declaration of 1964 and its later amendments. A waiver of informed consent and the Health Insurance Portability and Accountability Act, authorization requirement was granted as authorized by 45 CFR 46.116 (d) and 45 CFR 164.512 (i), respectively, as the research was determined to involve no more than minimal risk to the privacy of the individuals who are the subject of the protected health information. Data and study materials are not publicly available due to patient privacy protections. Reporting adheres to the Strengthening the Reporting of Observational Studies in Epidemiology (STROBE) statement [21].

### 2.2. Clinical Exposure and Outcomes Data

Data including demographics, comorbidities, clinical characteristics prior to mavacamten start and throughout treatment, and echocardiographic parameters were collected at the frequency recommended by the Risk Evaluation and Mitigation Strategy (REMS) program [22]. All data were independently extracted and verified by two trained abstractors. Any discrepancies were resolved by consensus review, ensuring reproducibility and consistency of measurements across the dataset. Dose adjustments were made per U.S. Prescribing Information prior to the April 2025 update. Doses of atrioventricular nodal blockade (AVNB) medications (beta blockers and nondihydropyridine calcium channel blockers) were ascertained at each patient visit through Week 108 of treatment. AVNB dosing was at the discretion of the treating clinician. Mavacamten doses and titrations were also captured along this time horizon. Resting transthoracic echocardiograms were performed at the frequency recommended by the REMS program [22] and as per the American Society of Echocardiography guidelines [23]. For patients in atrial fibrillation, echocardiographic measurements were averaged over at least five consecutive beats as recommended by the American Society of Echocardiography. Outcomes of interest included peak resting LVOT gradient, peak Valsalva LVOT gradient, LVEF, maximal LV wall thickness, and NYHA class at Week 0 (baseline, prior to mavacamten initiation) through Week 108. In this study, complete hemodynamic response to mavacamten was defined as resting LVOT gradient < 30 mm Hg, Valsalva LVOT gradient < 50 mm Hg, and LVEF > 50%. Additionally, hemodynamic response was also assessed using a modified EXPLORER-HCM definition of resting and Valsalva LVOT gradients < 30 mm Hg with LVEF > 50% and NYHA functional class I, reflecting complete symptomatic as well as physiologic response. We included both definitions to capture the clinical spectrum of response, as patients may demonstrate significant gradient reduction without full resolution of symptoms. Eligibility for septal reduction therapy (SRT) was assessed at Weeks 12 and 24 using an adapted guideline definition of persistence of NYHA functional class III or IV status and a dynamic LVOT gradient at rest or with provocation of ≥50 mm Hg [8]. Safety outcomes included data related to drug discontinuations and LVEF decrement to <50% while on mavacamten.

### 2.3. Statistical Analyses

Baseline characteristics were summarized as frequencies and percentages for categorical variables; continuous variables were reported as mean ± standard deviation (SD) for normally distributed data or median (interquartile range [IQR]) for non-normally distributed data. Normality of each continuous variable was assessed using the Shapiro–Wilk test and through visual inspection of histograms. The mean LVEF, maximal wall thickness, peak resting LVOT gradient, and peak Valsalva LVOT gradient were plotted over time along with 95% confidence intervals. Time to hemodynamic response to mavacamten was assessed using Kaplan–Meier analysis, with time zero designated as mavacamten start and patients right-censored at maximum follow-up (Week 108 or last available time point). The cumulative incidence of complete hemodynamic response was plotted over time along with 95% confidence bands. Next, to evaluate changes in NYHA class over time, the proportion of patients with NYHA class I, II, or III were displayed using stacked bar graphs at each time point. The proportion of patients experiencing ≥1 NYHA class improvement from baseline was also plotted over this time horizon. To evaluate changes in AVNB exposure, beta blocker total daily doses were first converted to metoprolol succinate equivalents (Supplementary Material). No dose conversions were performed for verapamil or diltiazem. The proportion of patients who underwent AVNB dose decrease, dose increase, or no dose change relative to baseline was then displayed using stacked bar graphs at each time point. Linear regression was used to assess the change in AVNB dose by class by week of therapy weighted by the number of patients at each time point. Finally, mavacamten dose exposure and titration over time was displayed at each time point using stacked bar graphs. All statistical analyses were performed using Stata/BE 18.0 (StataCorp, College Station, TX, USA) and were performed by the academic authors.

## 3. Results

### Baseline Characteristics

Over the study period, 163 patients were initiated on mavacamten, representing 9926 patient-weeks (190.8 patient-years) of mavacamten exposure. Median follow-up time was 60.0 (IQR 24.0, 96.0) weeks. Baseline characteristics are presented in Table 1.

Mean age of patients was 62.8 ± 13.7 years, 91 (55.8%) were female, and 136 (83.4%) were white. Patients had been diagnosed with HCM for a mean 8.9 ± 7.8 years, and 40 (24.5%) had a family history of HCM. Of the 124 (76.1%) who underwent genetic testing, 25 had pathogenic or likely pathogenic variants in sarcomere genes (MYBPC3 [myosin binding protein C3] = 16, MYH7 [myosin heavy chain 7] = 8, TNNT2 [cardiac type troponin T2] = 1). Cardiometabolic comorbidities were common as 99 (60.7%) had hypertension, 52 (31.9%) were overweight (body mass index [BMI] 25.0–29.9 kg/m^2^), 85 (52.1%) were obese (BMI > 30 kg/m^2^), 38 (23.3%) had a history of atrial fibrillation, and 26 (16.0%) had obstructive coronary artery disease. Forty-four patients (27.0%) had an implantable cardioverter defibrillator in place, and 14 (8.6%) had previously undergone SRT with either alcohol septal ablation (*n* = 4, 2.5%) or septal myectomy (*n* = 10, 6.1%). The majority (55.8%) were commercially insured. Nearly 20% lived further than 50 miles from the treatment center, 18.4% lived alone, and 16.6% did not identify a care partner.

Despite maximally tolerated guideline directed medical therapy with beta blocker alone (55.8%), nondihydropyridine calcium channel blocker alone (19.6%), beta blocker and nondihydropyridine calcium channel blocker (16.6%), or beta blocker and disopyramide (3.1%), the majority of patients remained severely symptomatic with NYHA III (54.0%) or IV (1.2%) functional class at baseline. Eight patients (4.9%) were on no background AVNB therapy due to medication intolerance. Mean baseline LVEF was 68.0 ± 5.7%. Patients were severely obstructed at rest (mean LVOT gradient 53.0 ± 36.7 mm Hg) and with Valsalva (79.6 ± 33.2 mm Hg).

In preparation for mavacamten initiation, patients on dual background HCM therapy with beta blocker and nondihydropyridine calcium channel blocker were adjusted to monotherapy, if possible, with preference toward beta blocker monotherapy. Mavacamten was started at the standard initiation dose of 5 mg in all except four (2.5%) patients. The reasons for initiation at the 2.5 mg dose in these patients included cross-titration with disopyramide (*n* = 2), concomitant treatment with amiodarone (*n* = 1) (possible potentiation of mavacamten effect), and prior intolerance of AVNB (*n* = 1).

At the time of this analysis, 153 patients had completed 12 weeks of therapy, and 33 had completed 108 weeks.

## 4. 108-Week Outcomes

### 4.1. Changes in LVOT Gradients, LVEF, LV Wall Thickness

**Figure 1:**
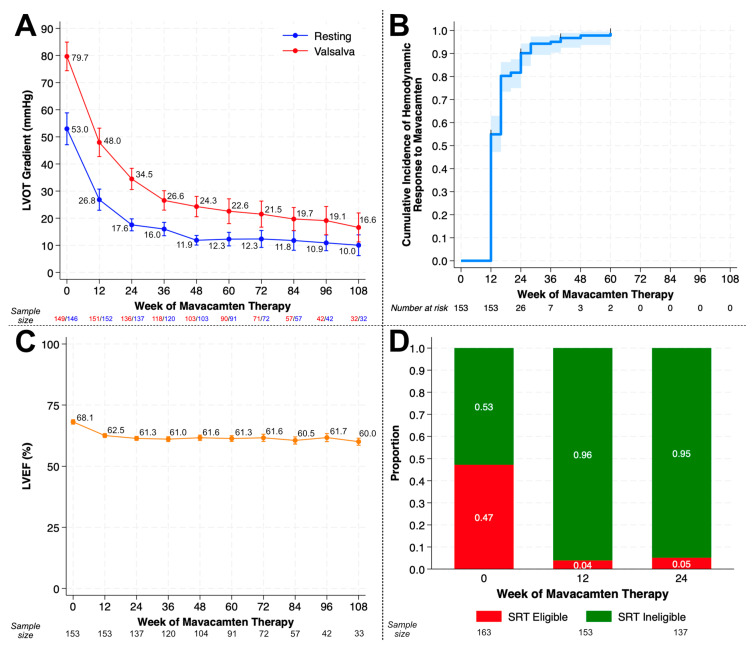
Left ventricular outflow tract (LVOT) gradients, hemodynamic response, and trajectory of left ventricular ejection fraction (LVEF) in patients on mavacamten. (**A**) Mean resting (blue) and Valsalva (red) LVOT gradients (mm Hg) at Weeks 0, 12, 24, 36, 48, 60, 72, 84, 96, and 108 of mavacamten therapy. Number of patients contributing to estimates at each time point are shown in blue (resting LVOT gradient) and red (Valsalva LVOT gradient). Error bars reflect 95% confidence intervals. (**B**) Complete hemodynamic response was defined as achievement of resting LVOT gradient < 30 mm Hg, Valsalva LVOT gradient < 50 mm Hg, and left ventricular ejection fraction > 50%. Patients were right censored at maximum follow up. Patients who did not complete >12 weeks of therapy were excluded from this analysis. (**C**) Mean LVEF at Weeks 0, 12, 24, 36, 48, 60, 72, 84, 96, and 108 of mavacamten therapy. Patients who did not complete >12 weeks of therapy were excluded from this analysis. Sample sizes contributing to estimates at each time point are shown. Error bars reflect 95% confidence intervals. (**D**) Proportion of patients who met adapted VALOR-HCM septal reduction therapy criteria at Weeks 0, 12, and 24. Abbreviations: SRT = septal reduction therapy.

Patients demonstrated rapid improvement in peak resting (53.0 mm Hg ± 36.7 to 10.0 mm Hg ± 11.0; *p* < 0.001) and Valsalva (79.7 mm Hg ± 33.2 to 16.6 mm Hg ± 15.4; *p* < 0.001) LVOT gradients after mavacamten initiation, and these gradient reductions were sustained through Week 108 (Figure 1A).

**Figure 2:**
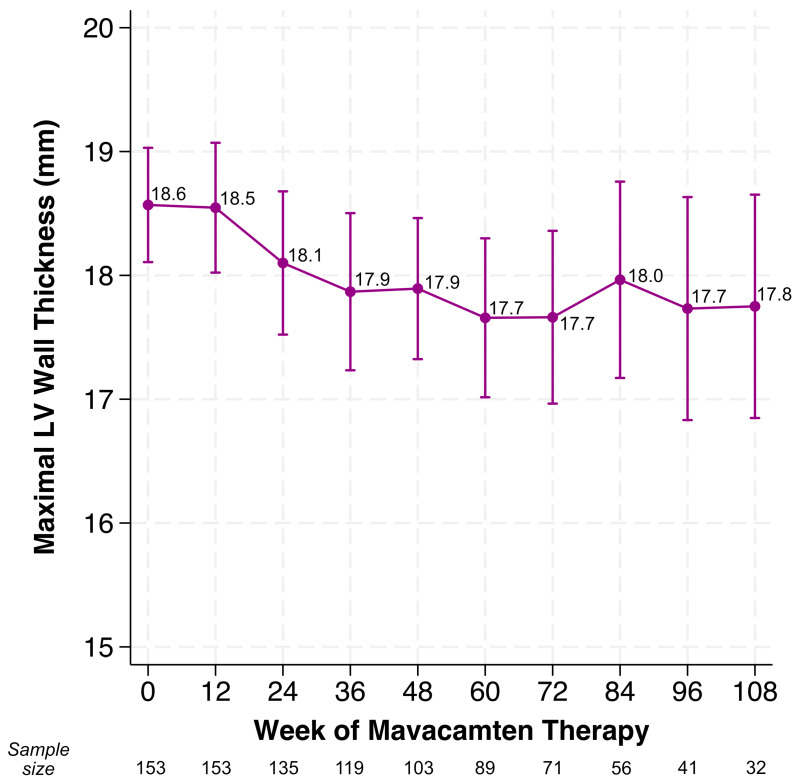
Changes in maximal left ventricular wall thickness in patients on mavacamten. Mean maximal left ventricular (LV) wall thickness at Weeks 0, 12, 24, 36, 48, 60, 72, 84, 96, and 108 of mavacamten therapy. Error bars reflect 95% confidence intervals.

Both mean LVOT gradients at rest and with Valsalva decreased to below the threshold criterion for LVOT obstruction (<30 mmHg) by Week 36 and remained below this level through Week 108. After restricting to only those who completed 12 weeks of mavacamten (i.e., completion of the initiation period as defined in the U.S. Prescribing Information) (*n* = 153), median time to achievement of complete hemodynamic response was 12 weeks, and over 90% of eligible patients achieved complete hemodynamic response by Week 24 (Figure 1B). Median time to hemodynamic response using the modified EXPLORER-HCM criteria was 36 weeks. Mean LVEF declined by 8.1% over the study period with the greatest change during the medication initiation period and minimal further change after Week 12 (Figure 1C). Using the VALOR-HCM SRT eligibility definition, the proportion of patients who remained eligible for SRT at Week 24 was 5%, a decrease from 47% at baseline (Figure 1D). Maximal LV wall thickness significantly decreased by 0.01 mm per week (*p* < 0.001; Figure 2).

### 4.2. Changes in NYHA Class

**Figure 3:**
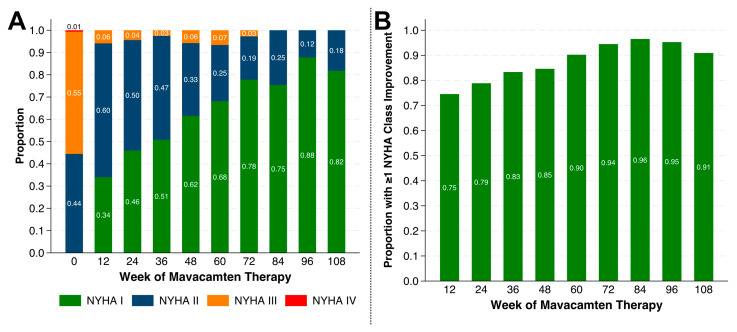
Changes in New York Heart Association (NYHA) class in patients on mavacamten. (**A**) Proportions of patients at NYHA I, II, III, IV functional classes at Weeks 0, 12, 24, 36, 48, 60, 72, 84, 96, and 108 of mavacamten therapy. (**B**) Proportion of patients who experienced >1 NYHA functional class improvement compared to their Week 0 NYHA class at Weeks 0, 12, 24, 36, 48, 60, 72, 84, 96, and 108.

At Week 0, 55.2% of patients (*n* = 90) had NYHA class III or IV functional limitation. By Week 12, 34% of patients were asymptomatic (i.e., had improved to NYHA I), and this proportion increased further to over half of patients by Week 36 and over 80% by Week 108 (Figure 3A). Compared with their baseline NYHA status, 75% of patients experienced >1 NYHA class improvement by Week 12 and 91% by Week 108 (Figure 3B).

### 4.3. Changes in Background Cardiovascular Medications

**Figure 4:**
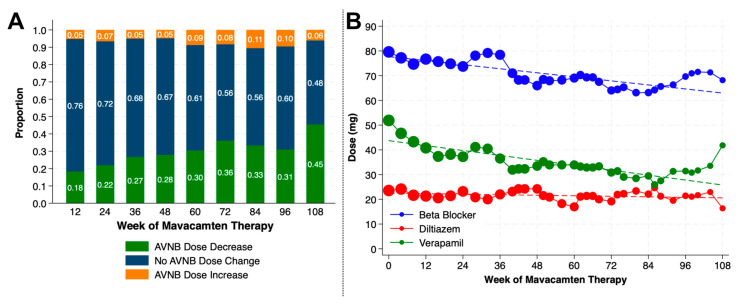
Changes in doses of atrioventricular nodal blocker (AVNB) background therapy. (**A**) AVNB doses at Weeks 12, 24, 36, 48, 60, 72, 84, 96, and 108 were calculated relative to AVNB doses at Week 0. Total daily doses of beta blockers were converted to approximate metoprolol succinate equivalents; no dose conversions were performed for verapamil and diltiazem. (**B**) Total daily dose in milligrams of beta blocker, verapamil, and diltiazem at the cohort level at Weeks 12, 24, 36, 48, 60, 72, 84, 96, and 108. Sizes of circles are proportional to number of patients at each time point. Dashed line represents trend line.

Mean systolic blood pressure for the cohort at Week 108 was 128.5 mm Hg (±12.9), and mean diastolic blood pressure was 78.7 mm Hg (±8.2). Thirty-five patients (21.5%) were newly initiated on antihypertensive medications during the study period. The most commonly used antihypertensive agents were angiotensin receptor blockers, with 42% on treatment by Week 108, and 21% were on mineralocorticoid receptor antagonists. By Week 12 of mavacamten therapy, 18% of patients were able to achieve a cumulative dose decrease in background AVNB compared to their baseline doses. This proportion steadily grew throughout the study period to 45% of patients with a dose decrease by Week 108 (Figure 4A). The majority of patients maintained the same AVNB total daily dose exposure throughout the study period. All five patients who were treated with disopyramide were weaned off by the Week 16 visit. At the total cohort level, the total daily doses of beta blocker (β = −0.14; 95% confidence interval [CI] −0.14, −0.13), verapamil (β = −0.17; 95% CI −0.17, −0.16), and diltiazem (β = −0.02; 95% CI −0.02, −0.02) significantly decreased over the study period (Figure 4B; *p* < 0.001 for all).

### 4.4. Mavacamten Dosing

**Figure 5:**
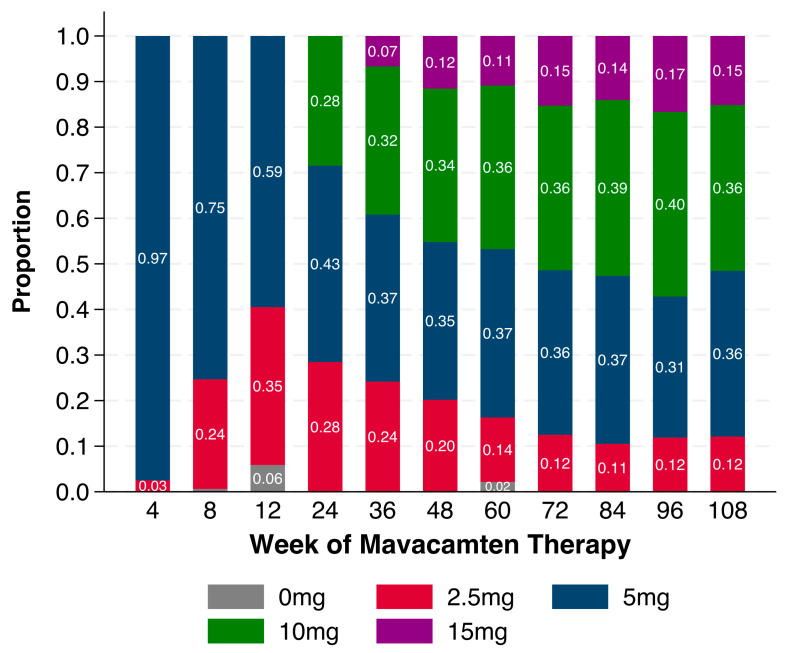
Mavacamten doses over 108 weeks of treatment. Proportions of patients on each dose of mavacamten at Weeks 12, 24, 36, 48, 60, 72, 84, 96, and 108.

Proportions of patients on each commercially available mavacamten dose at each time point are shown in Figure 5. Following the initiation period, 28% of patients were increased to the 10 mg daily dose by Week 24. From Weeks 48 through 108, 11–17% of patients were treated with the highest mavacamten dose of 15 mg daily, and similarly, 12–20% of patients were treated with the lowest dose of 2.5 mg daily. At Week 108, approximately one-third of patients were each treated with the 5 mg and 10 mg daily doses.

### 4.5. Temporary Interruption for LVOT Gradient and LVEF

All temporary interruptions for reduction in LVOT gradient < 20 mm Hg occurred at Week 8 (*n* = 7), and all patients were restarted on a lower mavacamten dose per U.S. Prescribing Information four weeks later. There were ten temporary interruptions for LVEF < 50% (6.1%), which occurred at Weeks 4 (*n* = 1, LVEF 45%), 8 (*n* = 2, LVEF 35–49%), 36 (*n* = 2, LVEF 44–49%), 56 (*n* = 1, LVEF 34%), 60 (*n* = 1, LVEF 48%), 72 (*n* = 2, LVEF 41–48%), and 84 (*n* = 1, LVEF 49%). None of these patients had symptomatic heart failure, all LVEFs improved to >50% with temporary cessation of mavacamten, and all were restarted on a lower mavacamten dose per U.S. Prescribing Information four weeks later. These individuals did not experience further complications during mavacamten therapy. One patient became pregnant on mavacamten, elected surgical abortion 1.5 months after drug discontinuation, and restarted mavacamten one month later.

### 4.6. Permanent Discontinuation

Mavacamten was discontinued in six patients (3.7%) during the study period. Two patients experienced complete hemodynamic response with LVOT gradient reduction; however, both ultimately underwent mitral valve replacement (one with concomitant septal myectomy) for severe primary mitral regurgitation. One patient elected to undergo myectomy after Week 48. Two patients experienced worsening atrial fibrillation without LVEF < 50%. One patient required hospitalization for management of stress cardiomyopathy with LVEF 35–40% at Week 58; LVEF recovered to 70% off mavacamten within 10 days. One patient died due to causes unrelated to HCM. No myocardial infarction, thromboembolic, or sustained ventricular arrhythmia events occurred during the study period.

## 5. Discussion

This study represents the largest and longest-in-duration single-center real-world experience to date of mavacamten initiation and corresponding outcomes in patients with symptomatic obstructive HCM. In comparison to previously published early real-world descriptions of mavacamten use, our study is unique in its large cohort size, demographic diversity, extended follow-up time, rigorous collection of hemodynamic and functional status endpoints, descriptions of background AVNB therapy and mavacamten dose trajectories, and comprehensive capture of safety data.

Our analysis extends several insights into the safe and effective use of mavacamten in the real world. Overall, we found that (1) patients treated with mavacamten experienced prompt LVOT gradient reduction with improvements in obstructive physiology continuing through Week 108; (2) complete hemodynamic response (resting LVOT gradient < 30 mmHg, Valsalva LVOT gradient < 50 mmHg, LVEF > 50%) occurs in more than half of patients by Week 12; (3) LVEF remained largely unchanged once patients completed the initiation phase; (4) 75% of patients experienced > 1 NYHA class improvement by Week 12; (5) background AVNB can be safely adjusted during mavacamten therapy; (6) 6% of patients experience temporary mavacamten discontinuation for LVOT gradient < 20 mm Hg or LVEF < 50% within the first 12 weeks when dosing is guided by the initial U.S. Prescribing Information; and (7) 1–3% of patients experience temporary discontinuations for LVEF < 50% after the first 12 weeks but are able to be restarted on therapy after normalization of LVEF.

We acknowledge that direct comparisons across real-world and clinical trial cohorts may introduce challenges; however, given the heterogeneity in patients with HCM, aligning these data can provide value to clinicians considering cardiac myosin inhibitor use. Compared with patients enrolled in the mavacamten treatment arm of the pivotal phase 3 EXPLORER-HCM [7] trial, our cohort was older, more demographically diverse, more symptomatic and functionally limited at mavacamten initiation, had more cardiovascular comorbidities, and had a longer average HCM duration. Longitudinal data from the Sarcomeric Human Cardiomyopathy Registry (SHaRe) registry has shown that the majority of HCM-related complications occur between ages 50 and 70 years [24], and our cohort largely fell into this age group. While we did not evaluate the association between mavacamten exposure and HCM-related adverse outcomes in this study, treatment with mavacamten did result in substantial improvements in obstructive physiology. As severe LVOT obstruction is an established independent risk factor for HF, future studies evaluating the association of mavacamten therapy with progression to HCM-related adverse outcomes like HF are an important next step. A small proportion of young patients with HCM were included in our study; 19.6% were <50 years old at mavacamten initiation. Similar age-matched cohort studies will be of critical importance to potentially position cardiac myosin inhibitors as disease-modifying therapy for individuals with obstructive HCM across the age spectrum.

The prevalence of cardiometabolic comorbidities was higher in our cohort when compared with patients treated with mavacamten in EXPLORER-HCM. Importantly, EXPLORER-HCM excluded individuals with documented obstructive coronary artery disease, defined as >70% stenosis in one or more epicardial coronary arteries, or a history of myocardial infarction. Sixteen percent of our cohort had coronary artery disease including individuals with prior myocardial infarction and percutaneous coronary intervention, and these individuals tolerated mavacamten without ischemia-related complications. Over half of the patients in our cohort were obese; data from the SHaRe registry has shown that obesity is associated with worse NYHA functional class and increased likelihood of obstructive physiology and adverse HCM-related outcomes [25]. As such, moving forward, using functional status assessment scales beyond NYHA class in patients on mavacamten may better elucidate the magnitude of symptom improvement related to resolution of LVOT obstruction versus the potential persistence of limitations related to excess and/or dysfunctional adiposity or other factors. While not a focus of the current study, 14 patients (8.6%) of our cohort were on incretin-based therapies at mavacamten initiation, which have been shown to be well-tolerated in a small cohort of patients on mavacamten [26]. Hemodynamic and functional status outcomes for this cohort is an important opportunity for future investigation.

Female patients composed the majority in our cohort, in contrast to other real-world cohorts [17,19]. Observational data have demonstrated that women with HCM are more likely to present with higher LVOT gradients [27] and higher symptom burden [28] but experience significant delay in HCM diagnosis and in the recognition of advanced symptoms that are largely driven by LVOT obstruction [29]. A prespecified post hoc analysis of sex from EXPLORER-HCM showed that treatment with mavacamten resulted in similar improvements in the primary and most secondary endpoints and greater improvements in health status in women compared with men [30], offering the potential for cardiac myosin inhibitor therapy to help close these persistent treatment gaps related to sex. Similarly, Black individuals with HCM are under-recognized and under-referred for therapies that relieve LVOT obstruction [31]. Rigorous real-world cohort studies like ours are critical to enhancing clinicians’ knowledge about mavacamten in order to facilitate broad and equitable access to this therapy beyond comprehensive HCM centers, which are not accessible to large populations of historically excluded individuals.

In EXPLORER-HCM and VALOR-HCM, background AVNB therapy was largely required to remain unchanged for the durations of the trials. However, in this real-world experience, we were able to safely modify background AVNB and disopyramide therapy during mavacamten initiation and titration. A considerable proportion of patients had indications for AVNB therapy beyond LVOT obstruction (e.g., atrial fibrillation, hypertension). As such, AVNB withdrawal was not an appropriate goal for every patient, but approximately 20–30% of patients were able to achieve a decrease in total daily AVNB dose when compared to baseline AVNB dose at each major safety visit. Our findings can support these important treatment decisions for patients as extended follow up data from long-term extension studies accrue [13].

Two patients in our cohort required permanent discontinuation of mavacamten for targeted treatment of underlying primary cardiovascular diseases that can mimic myocardial and structural derangements in HCM. Our experience highlights the importance of informed patient selection, comprehensive evaluation of LV hypertrophy including for HCM phenocopies, and characterization of mitral valve structure and function prior to initiation of cardiac myosin inhibitor therapy. One patient underwent myectomy after nearly a year of mavacamten treatment, emphasizing the treatment continuum for patients with obstructive HCM and role for continued shared decision-making during treatment with a novel therapy.

Our data demonstrate that there is a population of “early responders” to mavacamten therapy, i.e., those who achieve complete hemodynamic response in the early phases of drug initiation, and a small proportion of patients who may develop transient LVEF reduction during extended therapy. Taken together, these real-world data may be informative in guiding the timing of future echocardiographic safety protocols for cardiac myosin inhibitor therapy. We also show a small but significant change in maximal LV wall thickness over the course of treatment with mavacamten, supporting findings from MAVA-LTE [13] and highlighting the important opportunity to further investigate changes in myocardial structure related to cardiac myosin inhibitor treatment with serial echocardiography in the real world and to compare it to the left ventricular remodeling that occurs in the setting of afterload reduction after septal reduction therapy.

Limitations of our study include its tertiary referral and single-center nature, which could lead to selection bias toward patients with more severely symptomatic HCM and complex comorbidities. We are not able to make comparisons with patients treated with non-cardiac myosin inhibitor therapy, as this is a retrospective study of patients treated with mavacamten using data collected in routine clinical care. Follow-up was limited to 108 weeks for this analysis with variable follow-up periods due to the timing of mavacamten initiation. Future studies will report on longer-term outcomes. Our study reflects the experience of a single health system, and therefore generalizability to broader patient populations may be limited. Nonetheless, the findings provide important insights into the use of mavacamten in patients with obstructive hypertrophic cardiomyopathy in real-world practice. Broader multicenter studies will be essential to confirm these results across diverse clinical settings and patient populations, and to further inform implementation of this therapy.

## 6. Conclusions

Among adults with symptomatic obstructive HCM cared for at a high-volume comprehensive HCM center, treatment with mavacamten resulted in substantial and durable improvements in hemodynamics and functional status through 108 weeks. Altogether, our study provides numerous high-yield and clinically relevant insights into the real-world care of patients on the first-in-class cardiac myosin inhibitor, mavacamten, and supports further exploration in this era of molecularly targeted therapies in HCM.

## Figures and Tables

**Table 1:** Baseline Characteristics of Patients Initiated on Mavacamten.

Variable	(N = 163)
**Age at mavacamten start (years)**	
mean (±SD)	62.8 (13.7)
**Sex, n (%)**	
Female	91 (55.8)
Male	72 (44.2)
**Race, n (%)**	
White	136 (83.4)
Asian	8 (4.9)
Black or African American	6 (3.7)
Other	6 (3.7)
Not Reported	7 (4.3)
**Ethnicity, n (%)**	
Hispanic or Latino	4 (2.5)
**Insurance type, n (%)**	
Commercial (private)	91 (55.8)
Medicare/Medicare Advantage	71 (43.6)
Medicaid	1 (0.6)
**Care supports, n (%)**	
Has a care partner	136 (83.4)
Lives alone	30 (18.4)
**Distance from treating center, n (%)**	
≤10 miles	31 (19.0)
11–50 miles	100 (61.3)
51–200 miles	31 (19.0)
>200 miles	1 (0.6)
**Family history of HCM, n (%)**	40 (24.5)
**Genetic testing performed, n (%)**	124 (76.1)
Pathogenic or likely pathogenic variant present	26 (16.0)
*MYBPC3*	16 (9.8)
*MYH7*	8 (4.9)
*TNNT2*	1 (0.6)
*TPN11*	1 (0.6)
**Duration of HCM diagnosis (years)**	
mean (±SD)	8.9 (7.8)
**Medical history, n (%)**	
Hypertension	99 (60.7)
Coronary artery disease	26 (16.0)
Atrial fibrillation	38 (23.3)
Atrial fibrillation/flutter ablation	12 (7.4)
Implantable cardioverter defibrillator	44 (27.0)
Prior septal reduction therapy	14 (8.6)
Alcohol septal ablation	4 (2.5)
Septal myectomy	10 (6.1)
**New York Heart Association class, n (%)**	
II	73 (44.8)
III	88 (54.0)
IV	2 (1.2)
**Vital signs**	
BMI (kg/m^2^), mean (±SD)	30.9 (6.0)
Systolic blood pressure (mmHg), mean (±SD)	124.2 (14.8)
Diastolic blood pressure (mmHg), mean (±SD)	72.7 (9.1)
Heart rate (beats per minute), mean (±SD)	67.8 (12.4)
**Cardiac rhythm, n (%)**	
Sinus	145 (89.0)
Atrial-sensed, ventricular paced	4 (2.5)
Atrial-paced, ventricular-sensed	10 (6.1)
Atrial-paced, ventricular-paced	3 (1.8)
Ventricular-paced	1 (0.6)
**Echocardiographic parameters**	
LVEF (%), mean (±SD)	68.0 (5.7)
Maximal left ventricular wall thickness (mm), mean (±SD)	18.6 (2.9)
LVOT gradient at rest (mm Hg), mean (±SD)	53.0 (36.7)
LVOT gradient with Valsalva (mm Hg), mean (±SD)	79.7 (33.2)
Mitral regurgitation severity, n (%)	
Trace	21 (12.9)
Mild	68 (41.7)
Moderate	63 (38.7)
Severe	11 (6.8)
**Background HCM therapy, n (%)**	
Beta blocker alone	91 (55.8)
Nondihydropyridine calcium channel blocker alone	32 (19.6)
Beta blocker and nondihydropyridine calcium channel blocker	27 (16.6)
Beta blocker and disopyramide	5 (3.1)
None	8 (4.9)
**Starting mavacamten dose (mg), n (%)**	
2.5 mg	4 (2.5)
5.0 mg	159 (97.5)

Abbreviations: BMI = body mass index; HCM = hypertrophic cardiomyopathy; LVEF = left ventricular ejection fraction; LVOT = left ventricular outflow tract; MYBPC3 = myosin binding protein C, MYH7 = myosin heavy chain 7, SD = standard deviation, TNNT2 = cardiac type troponin T2.

## Data Availability

The data presented in this study are available on request from the corresponding author as the data are not publicly available due to patient privacy protections.

## Supplementary Materials

The following supporting information can be downloaded at: https://www.mdpi.com/article/10.3390/jcm14228181/s1, Table S1: Total Daily Beta-blocker Dose Conversion to Approximate Metoprolol Succinate Dose Equivalents.
