# Supplementary material for: Long-Term Real-World Outcomes of Mavacamten in Symptomatic Obstructive Hypertrophic Cardiomyopathy up to 108 Weeks
Source: J Clin Med. 2025 Nov 18;14(22):8181. doi: 10.3390/jcm14228181 (PMC12653594; doi:10.3390/jcm14228181)
Supplement: Supplementary file 1 [file jcm-14-08181-s001.zip › jcm-3934898-supplementary.pdf]

Table S1: Total Daily Beta-blocker Dose Conversion to Approximate Metoprolol Succinate Dose Equivalents

Drug	Factor Conversion
Atenolol	x 2
Bisoprolol	x 20
Carvedilol	x 4
Labetalol	x 0.5
Nadolol	x 1.25
Nebivolol	x 20
Propranolol	x 1.25
